# Diminished ability to integrate target stimuli with context during emotional recognition in individuals with broad autism phenotype

**DOI:** 10.3389/fpsyg.2022.934385

**Published:** 2022-10-06

**Authors:** Woo-Jin Cha, Jang-Han Lee

**Affiliations:** Department of Psychology, Chung-Ang University, Seoul, South Korea

**Keywords:** broad autism phenotype, autism spectrum disorder, weak central coherence theory, emotional recognition, congruency effect

## Abstract

Individuals with broad autism phenotype (BAP) have a tendency not to integrate emotional stimuli with the surrounding context. They have also shown different patterns and abilities in processing positive and negative emotions. This study aimed to examine whether the effect of context on target stimuli could vary depending on the type of target emotion in individuals with BAP. Based on the Broad Autism Phenotype Questionnaire (BAPQ) and Autism-Spectrum Quotient (AQ), 36 individuals with BAP and 33 healthy controls were selected. All the participants performed an overlap-emotional task consisting of six conditions: 2 (congruence: congruent and incongruent) × 3 (emotion: fearful, sad, and happy). Reaction time and accuracy were measured as dependent variables. The results revealed that the individuals with BAP showed no difference in reaction time between the condition of congruence and incongruence, but that the control group was faster to categorize facial expression on the condition of congruence than that of incongruence regardless of the type of target emotion. There were no differences between the two groups in any of the conditions with regard to accuracy. These findings indicate that individuals with BAP tend not to integrate target emotions with contextual information, a feature that could worsen the speed of emotional recognition in individuals with BAP. This study confirmed that the individuals with BAP have different cognition patterns in emotional recognition than the control group.

## Introduction

Autism spectrum disorder (ASD) is characterized by deficits in social interactions and restricted, repetitive patterns of behavior or interests (American Psychiatric Association, 2013). Deficits in social interaction represented by individuals with ASD could be affected by various causes, such as no interest in others or impairment of theory of mind, in which difficulty in emotional recognition can also have a significant effect on deficits in social interaction (Ingersoll, 2010). In particular, they cannot recognize negative emotions as easily as the general population (Farran et al., 2011; Santos et al., 2012).

The broad autism phenotype (BAP) has been defined as a trait qualitatively similar to autism spectrum disorder but with a mild level of symptoms. Individuals with BAP share a variety of autistic traits with the ASD group, including diminished social interaction and difficulty in emotional recognition (Jobe and White, 2007; Losh et al., 2009). As people express emotions and intentions not only through body posture but also their own facial expressions, the ability to read facial expressions is important for understanding others in their interactions with others (Adolphs, 2002; de Gelder, 2009; Frith, 2009). According to behavioral, neuroimaging and eye-tracking studies, individuals with BAP have a reduced ability to recognize emotions in other’s faces, resulted from different gaze fixation patterns and brain abnormalities (Dalton et al., 2007; Miu et al., 2012; Kadak et al., 2014).

Individuals with BAP are not helped by surrounding contextual information in emotional recognition. The face is rarely presented as a single entity but appears in situational contexts, and these contexts can impact the perception and evaluation of faces. Facial expressions can be recognized more accurately and quickly when facial expressions coincide with emotions in that context (Righart and de Gelder, 2008; Schwarz et al., 2013). Indeed, contextual information could have a greater impact on face recognition when facial stimuli are ambiguous (Masuda et al., 2008; Wieser and Brosch, 2012). If individuals with BAP had been assisted by surrounding contextual information, effective social interaction would have been possible by compensating for the decline in their emotional recognition. Conversely, individuals with BAP have a different cognitive pattern associated with diminished integration of the target with context; therefore, they tend not to integrate facial expressions with contextual emotions while performing a task wherein they have to identify facial emotions within contextual information (Hasegawa et al., 2014).

This different cognitive pattern of individuals with BAP has been explained by the weak central coherence theory (WCCT) (Happe and Frith, 2006). The WCCT, a representative theory that explains cognitive processing in ASD, points to a processing bias that prioritizes detailed and localized parts. Individuals with ASD organize each component independently compared to typically developing people that integrate and organize multiple components. Evidence of WCCT has been found in individuals with BAP regardless of the severity of their autistic traits (Briskman et al., 2001; Happe et al., 2001; Landry and Chouinard, 2016). This pattern of WCCT could explain the tendency not to integrate emotional faces with the emotional context demonstrated by individuals with BAP. In other words, the congruency effect, meaning that the ability to recognize facial expressions is increased when emotions of target and context are congruent relative to when they are incongruent, could not be found in individuals with BAP.

However, the cognitive pattern in individuals with BAP may vary depending on the type of emotion. The general population shows the congruency effect when recognizing facial emotions in context; in particular, when the happy face is presented, facial emotions could be recognized much better in the congruent condition than in the incongruent condition (Aguado et al., 2018, 2019). These results suggest that it took a long time to recognize happy faces in the incongruent condition because there was an attentional bias toward negative contexts such as being fearful or angry. Surprisingly, individuals with BAP not only have almost the same level of recognition for happy faces as the general population but also show an attentional bias to negative emotions, which means that the congruency effect may appear to be the same when recognizing happy faces in the BAP group (Milosavljevic et al., 2017; Wagner et al., 2020). In our study, we would identify whether the congruency effect would appear when individuals with BAP recognize a happy face, not a fearful or a sad one.

The present study would explore the ability of the BAP group to integrate targets with context in relation to fearful, sad, and happy emotions. The reason for choosing the fearful and sad expressions among the negative facial expressions is that individuals with autistic conditions have shown low awareness of fearful and sad emotions (Palermo et al., 2006; Poljac et al., 2013). Individuals with BAP have also demonstrated poor performance in recognizing angry expressions. However, we decided to exclude angry expressions because of the fact that differentiating between angry expressions and fearful expressions can be confusing not only for individuals with BAP but also for the general population (Juth et al., 2005). In addition, defects in the ability to recognize fearful and sad expressions have been found to be relevant to high levels of affective-interpersonal disturbances (Blair and Coles, 2000). These results indicate that fearful and sad emotions are highly related to defects in social interactions, as presented by individuals with BAP.

The aim of the present study is to investigate the cognitive and behavioral patterns of individuals with BAP when they recognize facial emotions surrounded by context. In order to determine this pattern, we aimed to elucidate whether the congruency effect could appear differently in recognizing facial emotion depending on the type of facial emotion in individuals with BAP. The research hypotheses are as follows: first, the congruency effect would be impaired in the BAP group compared to that in the control group. Second, in the BAP group, there would not be a congruency effect when they recognize a fearful or sad face, but when they recognize a happy face, the congruency effect would appear.

## Materials and methods

### Participants

Candidate participants were recruited through advertisements in online communities and Internet bulletin boards at several Universities in Seoul, Korea. Prior to the experiment, to screen for individuals with BAP, a total of 474 adults completed the Broad Autism Phenotype Questionnaire (BAPQ) (Hurley et al., 2007) and the Korean Version of the Autism Spectrum Quotient (AQ) (Ko et al., 2018). As in prior studies, the BAP group was selected from those who met the cut-off points, namely, an average BAPQ score of 3.15 or higher (the highest 25% of BAPQ score) and a total AQ score of 23 or higher (the highest 25% of AQ score). The control group included individuals with an average BAPQ score of 2.528 or less (the lowest 25% of BAPQ score) and a total AQ score of 16 or less (the lowest 25% of AQ score). Among those who met the above criteria, those who expressed willingness to participate in the experiment were selected as participants. The exclusion criteria for the present study were as follows: (1) diagnosis of other psychiatric disorders, (2) participation in any other pharmacological treatment, and (3) intellectual problems (i.e., a score below 70 on the K-WAIS-IV). Finally, 69 participants were sorted into the following two groups: (a) BAP group (*n* = 36) and (b) control group (*n* = 33). Specific demographic information is provided in the Results section.

### Questionnaires and measurement

#### The broad autism phenotype questionnaire

The BAPQ was used to assess autistic traits in the present study (Hurley et al., 2007). The BAPQ has been validated using the Korean version (Kim and Kim, 2022). The BAPQ is a 36-item self-reported scale scored using a 6-point Likert scale (1 = very rarely, 2 = rarely, 3 = occasionally, 4 = somewhat often, 5 = often, and 6 = very often). The BAPQ provides quantitative information associated with the diagnosis of autism spectrum disorder: aloof, pragmatic language, and rigid. Summary scores for the total were computed by averaging across all the 36 items, and summary scores for each scale were calculated by averaging across 12 items for each subscale. While the BAPQ was developed to assess the BAP in family members of individuals with ASD, this questionnaire could also measure the BAP in nonclinical samples of college students (Ingersoll et al., 2011). Before using the BAPQ in our study, its reliability and validity were confirmed using Korean samples. Cronbach’s α was 0.89 in the validation study and Cronbach’s α was 0.94 in the present study.

#### The autism-spectrum quotient

The AQ is a 50-item self-reported questionnaire developed to assess the degree of the five domains of autistic traits: social skill, communication, imagination, attention for detail, and attention switching (Baron-Cohen et al., 2001). The AQ has been validated using the Korean version (Ko et al., 2018). This questionnaire is scored using a four-point Likert scale (1 = strongly agree, 2 = agree, 3 = disagree, and 4 = strongly disagree). A score above 32 indicates clinically significant levels of autistic traits in the original scale, but a score above 23 could be used as an effective cutoff point for accurately classifying the maximum number of people with BAP in the Korean version of the AQ. Cronbach’s α was 0.85 in the validation study and was 0.89 in the present study.

#### The beck depression inventory-second edition

The BDI-II was used to compare and control the level of depression among the participants. The BDI-II was developed to assess the severity of subjective depression symptoms (Beck et al., 1996) and was validated in the Korean version (Lee et al., 2017). The BDI-II consists of 21 items related to cognitive and physical symptoms of depression during the previous week, and it is rated on a four-point Likert scale. A higher BDI-II score corresponds to a higher level of depression. Cronbach’s α was 0.89 in the validation study and 0.9 in the present study.

#### The beck anxiety inventory

The BAI was used to compare and control anxiety levels among the participants. The BAI was developed to measure anxiety levels (Beck et al., 1988) and was validated in Korean (Yook and Kim, 1997). It consists of 21 items related to physical and cognitive symptoms of anxiety during the previous week and is scored using a four-point Likert scale (0 = not at all, 1 = mild, 2 = moderate, and 3 = severe). Cronbach’s α was 0.91 in the validation study and 0.87 in the present study.

#### Korean-Wechsler adult intelligence scale-IV short form

The WAIS-IV was developed to measure intelligence and cognitive ability in individuals aged 16–90°years (Wechsler, 2008). In the present study, the K-WAIS-IV, standardized as the Korean version of the WAIS-IV, was used to confirm the difference in intelligence between groups that could influence task performance. The Arithmetic (AR) and Information (IN) subtests of the K-WAIS-IV were used, because it has been revealed that both subtests have the strongest correlation with full-scale intelligence quotient as a screening measure of intelligence (Hwang et al., 2012; Choe et al., 2014). Full-scale IQ could be estimated using regression equations [54.762 + (2.33 × AR) + (2.151 × IN)] (Choe et al., 2014).

### Behavioral task

#### The overlap-emotional task

**FIGURE 1:**
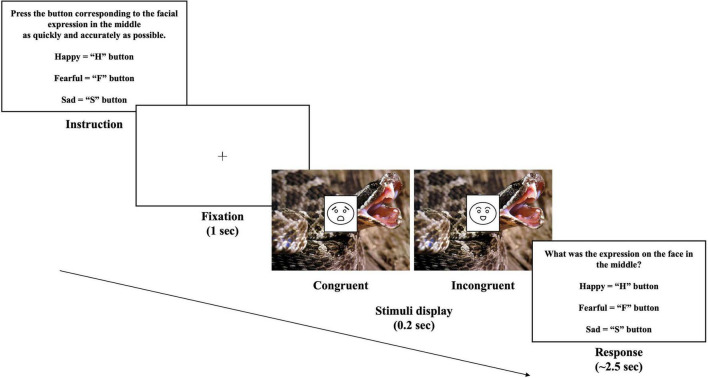
Trial example of the overlap-emotional Task. The stimuli were presented under the condition of congruence or incongruence. The original stimuli are unable to be reproduced for copyright reasons but are available upon request from the corresponding author.

The overlap-emotional task was performed to measure the accuracy and reaction time for categorizing the target emotion in context (see Figure 1). Contextual emotions might be the same or different from the target emotion (Righart and de Gelder, 2008). All pictures of the faces were used in full-blown facial expressions. The face stimuli were composed of 24 color face photographs balanced for gender, taken from “Extended Chae Lee Korean Facial Expressions of Emotions: ChaeLee-E” (Lee et al., 2013). The facial expressions included fearful (8), sad (8), and happy (8). The pictures of context consisted of 24 pictures; fearful (8), sad (8), and happy (8) scenes taken from the International Affective Picture System (Lang et al., 1997), the Open Affective Standardized Image Set (Kurdi et al., 2017), and the Geneva Affective Picture Database (Dan-Glauser and Scherer, 2011). Supplementary Table 1 shows which pictures are used. To validate the valence and arousal of the pictures displaying fearful, sad, and angry expressions, 15 graduate students rated the relevance, arousal, and valence of the emotions using a seven-point Likert scale (0 = Not at all, 6 = Extremely). Notably, there was no significant difference in the arousal of emotions for either type of stimulus. Supplementary Table 2 shows the ratings for fearful, sad, and angry emotions of the pictures.

The participants were required to focus on the center of the target-context stimulus when they performed the task. The facial stimuli were placed at the center of the scene to avoid saccades. Emotional scenes were carefully edited to prevent the facial stimuli from covering a critical part of the scene related to emotion. The width and height of the target (emotional faces) and context (emotional scenes) were 7 × 9, and 35 cm × 26 cm, respectively. After fixation was presented for 1 s, the stimuli were presented only for 0.2 s and disappeared. The participants were required to answer what the emotion of the target was as quickly and accurately as possible while ignoring the emotions of the surrounding context. When the targets were presented as fearful, sad, or happy, the answers were the F, S, or H, buttons, respectively. They were instructed to react with their hand on the keyboard to respond as quickly as possible. If a participant failed to respond within 2.5 s, it would be an incorrect answer and moved on to the next question. Reaction time was rated from the point at which the stimulus was presented. This task consisted of eight blocks, with each block composed of 48 trials. The total number of congruence and incongruence conditions was the same to avoid predictability (FF, SS, and HH, each of eight trials per block; FS, FH, SF, SH, HF, and HS, each of four trials per one block), and the order of each trial was presented randomly.

### Apparatus

The participants performed the overlap-emotional task at a viewing distance of approximately 60 cm from a 15-inch monitor with a resolution of 1920 × 1080. The task was produced using the PsychoPy software (version 3.0) forf Windows (Peirce, 2009), which provided the information about the participant’s responses, such as reaction time and accuracy.

### Procedure

This study was performed in accordance with the principles of the Declaration of Helsinki. The participants visited the laboratory and listened to a brief introduction to the experiment and their rights as research participants. All the participants took part in the experiment upon signing an informed consent form approved by the Institutional Review Board of Chung-Ang University (No. 1041078-202008-HR-216-01). After they signed the informed consent form, they were interviewed using the brief version of the K-WAIS-IV and completed the self-reports, the BDI-II and BAI. Afterward, the participants were encouraged to sit comfortably about 60 cm away from the monitor. Prior to the main experiment, all the participants conducted a preliminary experiment that required them to categorize emotions corresponding to 12 facial expressions without a time limit. The emotions that the participants should categorize in the preliminary experiment were fearful, sad, and happy. All the participants were instructed to categorize the emotions as quickly and accurately as possible and had sufficient practice trials before the main experiment to fully understand how to perform. The participants were able to relax as much as they wanted between blocks. After all the experiments, the participants were debriefed on the aim of the experiment and received a fixed reward. The duration of the experiment was approximately 30 min per person.

### Data analysis

For data analysis, one-way analysis of variance (ANOVA) and chi-square test were performed to analyze differences in group characteristics between the broad autism phenotype and control groups. A 2 (group: BAP and control) × 2 (congruence: congruent and incongruent) × 3 (emotion of target: fearful, sad, and happy) mixed-design ANOVA was conducted to analyze the data. If the assumption of sphericity or homogeneity of variance was violated when Mauchly’s Test of Sphericity and Levene’s Test of Equality of Error Variances were checked, a Greenhouse-Geisser correction was applied. When the ANOVA showed significant effects, Bonferroni’s *post hoc* tests were conducted. To confirm the relationship between task performance (reaction time and accuracy) and clinical scales indicating autistic traits, we performed a correlation analysis (Pearson’s *r*). When we performed the correlation analysis, we made congruency scores of reaction time and accuracy and confirmed the relationship between congruency scores and autistic traits. The values of the congruency score are calculated using the difference in reaction time (RT) and the difference in error rate (ER) (congruency score of reaction time = RT in incongruent condition–RT in congruent condition, congruency score of accuracy = ER in incongruent condition–ER in congruent condition). A higher congruency score indicates that the participants are more influenced by the context. A statistical analysis was conducted using SPSS (version 26.0) for Windows.

After confirming the effect of mixed design ANOVA and correlation analysis, we also computed Bayes factors (BF_10_) to quantify alternative and null hypothesis strengths (Rouder and Morey, 2009; Morey and Rouder, 2022). Unlike frequentist hypothesis tests, Bayes factors make it possible to report evidence in favor of the null hypothesis rather than failing to reject the null hypothesis, so Bayes factors could complement the *p*-value from the null-hypothesis significance test. A Bayesian analysis was conducted using the JASP software (JASP Team, 2019).

To establish the appropriate sample size, the program G*Power 3.1.9.4 (University of Dusseldorf, Dusseldorf, Germany) was employed prior to our study. A total sample size of 28 people was estimated to be an adequate number for a mixed-design ANOVA, with an alpha error probability of 0.05 (two-tailed), a power of 0.95, and a medium effect size (*η_*p*_^2^* = 0.25).

To analyze the data, the reaction time was calculated as the mean for the correct response, and the accuracy was measured as the error rate, sensitivity, and response criteria. Sensitivity refers to the level at which target stimuli are distinguished sensitively from non-target stimuli, and the response criterion refers to bias when selecting specific target stimuli (Macmillan and Creelman, 2005). The values of the sensitivity and response criteria are calculated using hit rate (*H*) and false alarm (*FA*) {sensitivity = z(*H*)–z(*FA*); response criteria = –1/2 × [z(*H*) + z(*FA*)]}. Hit rate is the degree to which a specific stimulus is accurately detected when it appears (e.g., the hit rate of a fearful face indicates the degree to which a fearful face is accurately detected when it appears). False alarm refers to the degree to which participants responded incorrectly that a specific stimulus appeared even though the specific stimulus did not appear (e.g., false alarm of a fearful face refers to the degree to which participants responded that a fearful face appeared even though a sad or happy face appeared). A higher sensitivity score indicates that a participant could distinguish a particular emotion well. Response criteria close to zero indicate that there is no bias in selecting a particular emotion. Response criteria lower than 0 indicate a bias to respond that a particular emotion exists even though it does not. Response criteria greater than 0 indicate a bias to respond that a particular emotion does not exist even though it does. Response criteria and sensitivity should be measured to calculate accuracy, because incorrect answers could result not only from mistakes but also from biases wherein a particular response is selected continuously. Calculating the sensitivity and response criteria allows the mistakes and bias of participants to be considered simultaneously.

When the incongruent conditions were analyzed, combinations of fearful and sad were excluded from the analysis. This is because although “fearful” and “sad” are different emotions, sad targets (e.g., crying face) in fearful contexts (e.g., scary situation) are a sufficiently matched condition. Therefore, we decided that considering the sad face presented in the fearful context as an incongruent condition would not have a clear result and decided to exclude the combinations of fear and sadness from the analysis. We presented the results including the combinations of fearful and sad emotions in the Supplementary material (Supplementary Figure 2; Supplementary Tables 3, 4). Considering that the fearful and sad emotions were assessed as negative valence, we also conducted a 2 (group: BAP and control) × 2 (congruence: congruent and incongruent) × 2 (valence of target: negative and positive) mixed-design ANOVA and presented the results in the Supplementary material (Supplementary Tables 5, 6).

Of all the participants, six were removed from the analysis: one participant with missing data due to task error and five participants whose error rate in the task was more than 2 SDs of the average. Ultimately, 32 individuals with BAP and 31 healthy controls were included in the final sample of the present study.

## Results

### Group characteristics

Table 1 shows the demographic and clinical characteristics of the participants acquired from the results of the one-way ANOVA and the chi-square test. Group characteristics were analyzed by age, sex, estimated IQ (arithmetic, information), characteristics of autism, depression, and anxiety.

**TABLE 1:** Demographic and clinical characteristics for each group.

Measure	BAP (*n* = 32)	Control (*n* = 31)	Test statistics		
			
			*df*	*F*/*χ 2*	*p*
Age (years)	23.500 (2.463)	23.130 (2.156)	1, 61	0.404	0.528
Sex (male/female)	17/15	12/19	1	1.317	0.251
IQ	108.775 (7.859)	109.320 (10.859)	1, 61	0.052	0.820
BAPQ	3.617 (0.336)	2.184 (0.224)	1, 61	398.532	**< 0.001**
Aloof	2.269 (0.561)	3.904 (0.561)	1, 61	133.663	**< 0.001**
Pragmatic language	2.118 (0.219)	3.255 (0.407)	1, 61	188.706	**< 0.001**
Rigid	2.145 (0.378)	3.693 (0.506)	1, 61	187.908	**< 0.001**
AQ	28.090 (3.402)	11.320 (2.833)	1, 61	450.654	**< 0.001**
Social skill	1.81 (0.946)	6.470 (1.367)	1, 61	246.188	**< 0.001**
Communication	0.480 (0.677)	4.910 (2.053)	1, 61	130.036	**< 0.001**
Imagination	1.520 (1.029)	4.190 (1.891)	1, 61	48.062	**< 0.001**
Attention for detail	3.450 (2.158)	5.030 (1.858)	1, 61	9.717	**0.003**
Attention switching	4.060 (1.526)	7.500 (1.437)	1, 61	84.682	**< 0.001**
BDI-II	6.220 (6.173)	4.420 (4.537)	1, 61	1.729	0.193
BAI	5.310 (5.064)	4.710 (4.907)	1, 61	0.230	0.633

Mean (standard deviation). BAP, broad autism phenotype group; Control, control group; Age, years, IQ, intelligence quotient estimated by brief version of Korean-Wechsler Adult Intelligence Scale-IV; BAPQ, Broad Autism Phenotype Questionnaire; AQ, Autism Spectrum Quotient; BDI-II, Beck Depression Inventory-II; and BAI, Beck Anxiety Inventory. Bold value indicates that the value is statistically significant.

According to the selection criteria, there were significant main effects on average BAPQ and total AQ score. These results indicated that the groups were properly divided according to their autistic traits. There were no significant differences in age, sex, or estimated IQ between the groups. There were also nonsignificant differences in clinical measures such as BDI-II and BAI. These facts indicate that the results of the present study were not affected by any other demographic or psychological factor.

### Reaction time

To examine the speed of recognition of faces with emotions, a 2 × 2 × 3 mixed-design ANOVA on reaction time was performed (see Table 2 and Supplementary Figure 1).

**TABLE 2:** Mean (SD) of reaction time (in s) under each emotion and congruence condition for the groups.

Facial expression	Context	BAP (*n* = 32)	Control (*n* = 31)	Test statistics			
				
				*df*	*F*	*p*	*BF*_10_
Fearful	Con: Fearful	0.780 (0.331)	0.542 (0.165)	1, 61	12.932	**< 0.001**	44.648
	Incon: Happy	0.779 (0.298)	0.582 (0.241)	1, 61	8.323	**0.005**	7.666
Sad	Con: Sad	0.786 (0.289)	0.581 (0.191)	1, 61	10.962	**0.002**	21.234
	Incon: Happy	0.742 (0.273)	0.613 (0.249)	1, 61	3.850	0.054	1.280
Happy	Con: Happy	0.610 (0.246)	0.471 (0.168)	1, 61	6.792	**0.012**	4.174
	Incon: Fearful or Sad	0.634 (0.263)	0.489 (0.170)	1, 61	6.706	**0.012**	4.041

Mean (standard deviation). BAP, broad autism phenotype group; Control, control group; Con, congruent; and Incon, incongruent. Congruent is a condition in which emotions of target and context are the same. Incongruent is a condition in which emotions of target and context are not the same. Bold value indicates that the value is statistically significant.

**FIGURE 2:**
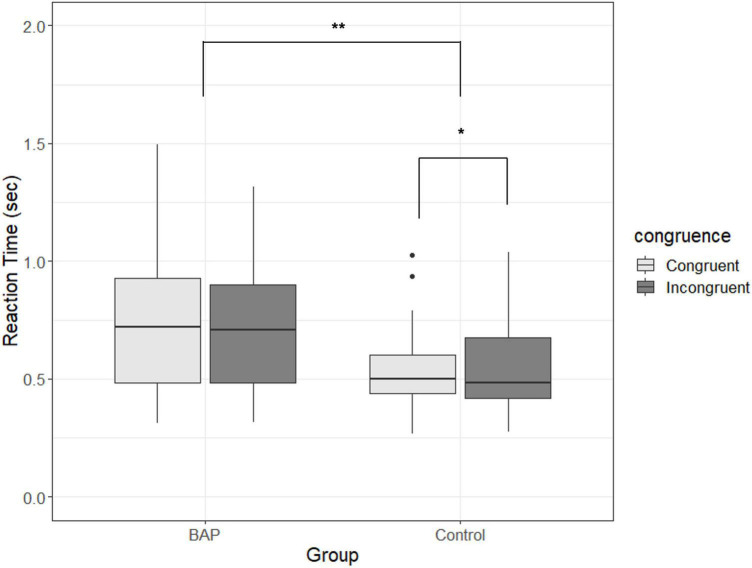
Comparison of reaction time for congruence in the broad autism phenotype (BAP) and control groups. Congruent is a condition in which emotions of target and context are the same; incongruent is a condition in which emotions of target and context are not the same. Box plots show the median as the middle box line, the first quartile (Q1) and third quartile (Q3) as box edges (denoting the interquartile range, IQR), whiskers as the minimum/maximum points and outliers based on thresholds < Q1–1.5 (IQR) or > Q3+1.5 (IQR). BAP, broad autism phenotype group; Control, control group. *p < 0.05. **p < 0.01.

There was no significant three-way interaction effect [*F* (1.847, 112.657) = 2.146, *p* = 0.126, *η_*p*_^2^* = 0.034, BF_10_ < 0.03]. However, there was a significant two-way interaction effect on group × congruence [*F*(1, 61) = 4.653, *p* = 0.035, *η_*p*_^2^* = 0.071, BF_10_ = 3.904]. To confirm the significant two-way interaction effect in terms of the purpose of our study, the main effects of congruence were analyzed in each group. While there was no difference between the congruent and incongruent conditions in the BAP group [*F*(1, 31) = 0.306, *p* = 0.584, *η_*p*_^2^* = 0.01, BF_10_ = 0.287], there was a significant main effect between congruent and incongruent conditions in the control group [*F*(1, 30) = 6.368, *p* = 0.017, *η_*p*_^2^* = 0.175, BF_10_ = 2.871]. That is, while the control group showed faster reaction times when the emotions of the target and context are congruent rather than incongruent, the BAP group showed no difference in reaction time between the congruent and incongruent conditions regardless of the type of emotion (refer to Figure 2).

**FIGURE 3:**
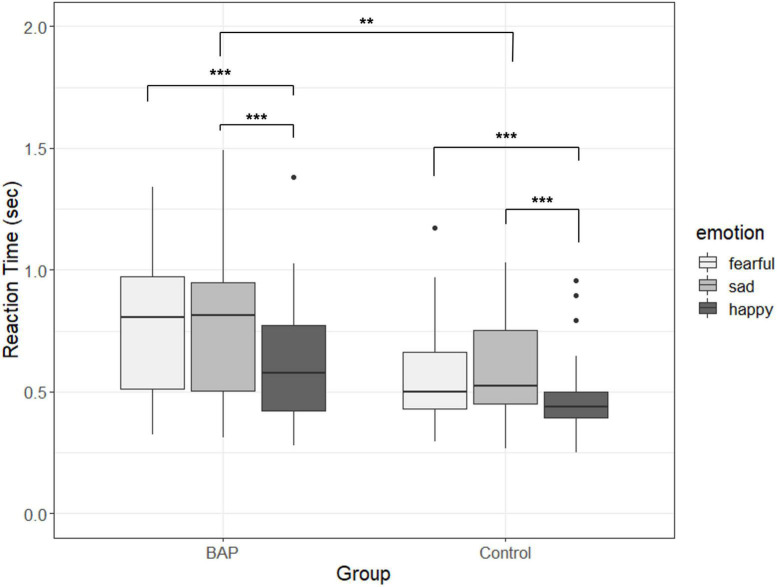
Comparison of reaction time for emotion in the broad autism phenotype (BAP) and control groups. Box plots show the median as the middle box line, the first quartile (Q1), and third quartile (Q3) as box edges (denoting the interquartile range, IQR), whiskers as the minimum/maximum points and outliers based on thresholds < Q1–1.5 (IQR) or > Q3+1.5 (IQR). BAP, broad autism phenotype group; Control, control group. **p < 0.01. ***p < 0.001.

There was also a significant two-way interaction in group × emotion [*F*(1.907, 116.348) = 3.295, *p* = 0.043, *η_*p*_^2^* = 0.051, BF_10_ = 0.043]. When *post hoc* tests by the Bonferroni correction were performed to confirm the significant emotion difference in each group, both groups recognized happy emotions faster than other emotions. Additionally, the BAP group recognized fearful emotions slower than sad emotions. In the control group, the converse was true; they recognized sad emotions slower than fearful emotions (refer to Figure 3).

There was a significant main effect of group [*F*(1, 61) = 9.17, *p* = 0.004, *η_*p*_^2^* = 0.131, BF_10_ = 6.743]. This main effect reflected the fact that the BAP group recognized the facial expressions slower than the control group. There was also a significant main effect of emotion [*F*(1.907, 116.348) = 45.618, *p* < 0.001, *ηp2* = 0.428, BF_10_ > 30]. The *post hoc* tests revealed that happy faces could be recognized faster than other emotions, but that there was no significant difference between fearful and sad faces.

In sum, the BAP group recognized emotions more slowly than the control group. In particular, when recognizing the emotion of the target, there was no difference between the congruent and incongruent conditions in the BAP group, while the control group could recognize the emotion of the target more quickly in the congruent than in the incongruent condition.

### Accuracy

#### Error rate

To examine the accuracy of recognition of emotional faces, a 2 × 2 × 3 mixed-design ANOVA on the error rate was performed (refer to Table 3).

**TABLE 3:** Mean (SD) of error rate under each emotion and congruence condition for the groups.

Facial expression	Context	BAP (*n* = 32)	Control (*n* = 31)	Test statistics			
				
				*df*	*F*	*p*	*BF*_10_
Fearful	Con: Fearful	5.273 (4.098)	4.503 (5.020)	1, 61	0.447	0.506	0.311
	Incon: Happy	5.599 (6.827)	6.586 (8.456)	1, 61	0.261	0.611	0.287
Sad	Con: Sad	7.292 (6.673)	6.653 (7.520)	1, 61	0.127	0.723	0.271
	Incon: Happy	8.203 (7.520)	6.452 (6.440)	1, 61	0.983	0.325	0.389
Happy	Con: Happy	3.190 (3.778)	3.763 (3.544)	1, 61	0.385	0.537	0.303
	Incon: Fearful or Sad	3.971 (3.901)	4.167 (3.916)	1, 61	0.039	0.843	0.261

Mean (standard deviation). BAP, broad autism phenotype group; Control, control group; Con, congruent; and Incon, incongruent. Congruent is a condition in which emotions of target and context are the same. Incongruent is a condition in which emotions of target and context are not the same.

There was no significant three-way interaction [*F*(1.783, 108.769) = 1.067, *p* = 0.341, *η_*p*_^2^* = 0.017, BF_10_ < 0.03]. Unlike reaction time, there was no significant interaction effect on group × congruence [*F*(1, 61) = 0.015, *p* = 0.902, *η_*p*_^2^* = 0, BF_10_ = 0.111]. There was also no significant two-way interaction in group × emotion [*F*(1.97, 120.195) = 0.662, *p* = 0.515, *η_*p*_^2^* = 0.011, BF_10_ = 0.041].

There was a significant main effect of emotion [*F*(1.97, 120.195) = 10.617, *p* < 0.001, *η_*p*_^2^* = 0.148, BF_10_ > 30]. *Post hoc* tests by Bonferroni correction revealed that the happy emotion could be recognized more accurately than the other emotions, and that there was no significant difference between the fearful and sad emotions. There was a marginally significant tendency for congruence [*F*(1, 61) = 4.011, *p* = 0.05, *η_*p*_^2^* = 0.062, BF_10_ = 1.129]. There was no significant main effect on group [*F*(1, 61) = 0.054, *p* = 0.817, *ηp2* = 0.001, BF_10_ = 0.263], indicating that the BAP group recognized facial expression as accurately as the controls.

These findings showed that the BAP and control groups could recognize happy faces more accurately than the other emotions. Moreover, the BAP group recognized the facial expressions as accurately as the controls.

#### Sensitivity

To examine the level of discrimination of the target emotion, a 2 × 2 × 3 mixed-design ANOVA on sensitivity was performed (refer to Table 4).

**TABLE 4:** Mean (SD) of sensitivity under each emotion and congruence condition for the groups.

Facial expression	Context	BAP (*n* = 32)	Control (*n* = 31)	Test statistics			
				
				*df*	*F*	*p*	*BF*_10_
Fearful	Con: Fearful	−0.051 (1.357)	0.053 (1.897)	1, 61	0.063	0.802	0.264
	Incon: Happy	−0.063 (1.414)	0.065 (1.774)	1, 61	0.101	0.752	0.268
Sad	Con: Sad	−0.321 (1.900)	0.331 (1.083)	1, 61	2.773	0.101	0.821
	Incon: Happy	−0.285 (2.217)	0.294 (0.827)	1, 61	1.859	0.178	0.562
Happy	Con: Happy	−0.054 (1.763)	0.055 (1.418)	1, 61	0.073	0.789	0.265
	Incon: Fearful or Sad	−0.137 (1.745)	0.142 (1.511)	1, 61	0.458	0.501	0.312

Mean (standard deviation). BAP, broad autism phenotype group; Control, control group; Con, congruent; and Incon, incongruent. Congruent is a condition in which emotions of target and context are the same. Incongruent is a condition in which emotions of target and context are not the same.

There was a nonsignificant three-way interaction effect [*F*(1.538, 93.812) = 0.198, *p* = 0.762, *η_*p*_^2^* = 0.003, BF_10_ < 0.03]. Unlike reaction time, there was no significant interaction effect on group × congruence [*F*(1, 61) = 0.027, *p* = 0.871, *η_*p*_^2^* = 0, BF_10_ = 0.027]. There was also a nonsignificant two-way interaction effect on group × emotion [*F*(1.882, 114.78) = 1.407, *p* = 0.249, *η_*p*_^2^* = 0.023, BF_10_ < 0.03]. There was no significant main effect of group [*F*(1, 61) = 0.907, *p* = 0.345, *η_*p*_^2^* = 0.015, BF_10_ = 0.377], indicating that the BAP group recognized the facial expressions as accurately as the control group.

These findings showed that both the BAP and control groups showed similar ability levels in discriminating the emotions sensitively regardless of the type of emotion and congruence condition. Especially, there was no congruency effect on either the BAP group or the control group.

#### Response criteria

To examine the bias in selecting particular emotions that can impact the accuracy of facial expression recognition, a 2 × 2 × 3 mixed-design ANOVA on response criteria was performed (refer to Table 5).

**TABLE 5:** Mean (SD) of response criteria under each emotion and congruence condition for the groups.

Facial expression	Context	BAP (*n* = 32)	Control (*n* = 31)	Test statistics			
				
				*df*	*F*	*p*	*BF*_10_
Fearful	Con: Fearful	−0.001 (0.553)	0.001 (0.619)	1, 61	0.000	0.986	0.257
	Incon: Happy	−0.061 (0.681)	0.062 (0.524)	1, 61	0.643	0.426	0.337
Sad	Con: Sad	−0.090 (0.678)	0.093 (0.541)	1, 61	1.393	0.243	0.462
	Incon: Happy	0.020 (0.630)	−0.021 (0.414)	1, 61	0.093	0.761	0.267
Happy	Con: Happy	−0.044 (0.593)	0.045 (0.626)	1, 61	0.334	0.566	0.296
	Incon: Fearful or Sad	−0.051 (0.601)	0.053 (0.567)	1, 61	0.504	0.480	0.318

Mean (standard deviation). BAP, broad autism phenotype group; Control, control group; Con, congruent; and Incon, incongruent. Congruent is a condition in which emotions of target and context are the same. Incongruent is a condition in which emotions of target and context are not the same.

There was no significant three-way interaction effect [*F*(2, 122) = 0.682, *p* = 0.501, *η_*p*_^2^* = 0.011, BF_10_ < 0.03]. There was no significant interaction effect on group × congruence [*F*(1, 61) = 0.231, *p* = 0.633, *η_*p*_^2^* = 0.004, BF_10_ = 0.05]. There was also a nonsignificant two-way interaction effect on group × emotion [*F*(2, 122) = 0.018, *p* = 0.982, *η_*p*_^2^* = 0, BF_10_ < 0.03]. There was no significant main effect of group [*F*(1, 61) = 3.316, *p* = 0.074, *η_*p*_^2^* = 0.052, BF_10_ = 1.027].

These findings indicate that there was no significant difference between the BAP and control groups with respect to bias in selecting specific emotions. In addition, both groups showed response criteria close to zero for all the conditions, which means that both groups were not biased in selecting specific emotions when they chose the target emotion. In summary, the two groups expressed a similar level of accuracy for the recognition of facial expressions without being affected by bias in selecting specific emotions.

### Correlation analysis

Pearson’s correlation analysis was conducted to examine the relationship between behavioral parameters (reaction time, error rate, sensitivity, and response criteria) and autistic traits (BAPQ and AQ).

Reaction time was positively correlated with the overall and subtype scores on the BAPQ. Reaction time was also positively correlated with the overall and subtype scores on the AQ except for attention for detail. The reaction time in the congruent condition was positively correlated with all the subtypes of BAPQ and AQ except for attention for detail. The reaction time in the incongruent condition was positively correlated with the overall score and aloof subtype of the BAPQ. The reaction time in the incongruent condition was also positively correlated with the overall and subtype scores on the AQ except for attention for detail (refer to Table 6). There was no significant correlation between autistic traits and error rate, sensitivity, and response criteria (Supplementary Tables 7–9). There were some significant correlations between autistic traits and congruency score on RT, but the raw data of reaction time under each condition was found to be more related to autistic traits than congruency score (Supplementary Table 10).

**TABLE 6:** Correlation analysis between reaction time and score on broad autism phenotype questionnaire (BAPQ) and autism-spectrum quotient (AQ).

	RT-Total			RT-Con			RT-Incon		
	
	*r*	*p*	*BF*_10_	*r*	*p*	*BF*_10_	*r*	*p*	*BF*_10_
BAPQ	0.324	**< 0.001**	4.186	0.358	**< 0.001**	9.223	0.283	**0.025**	1.851
Aloof	0.319	**0.011**	3.750	0.364	**0.003**	10.558	0.267	**0.034**	1.401
Pragmatic language	0.265	**0.036**	1.361	0.286	**0.023**	1.983	0.238	0.060	0.888
Rigid	0.276	**0.028**	1.644	0.302	**0.016**	2.647	0.245	0.053	0.981
AQ	0.370	**< 0.001**	12.349	0.399	**0.001**	26.817	0.334	**0.008**	5.158
Social skill	0.317	**0.011**	3.573	0.329	**0.009**	4.620	0.298	**0.018**	2.457
Communication	0.279	**0.027**	1.732	0.299	**0.017**	2.532	0.253	**0.045**	1.112
Imagination	0.312	**0.013**	3.224	0.354	**0.004**	8.208	0.263	**0.037**	1.302
Attention for detail	0.223	0.079	0.707	0.237	0.061	0.871	0.204	0.110	0.551
Attention switching	0.282	**0.025**	1.828	0.307	**0.014**	2.975	0.251	**0.047**	1.074

RT, reaction Time; Con, congruent; Incon, incongruent; BAPQ, Broad Autism Phenotype Questionnaire; AQ, Autism Spectrum Quotient. Congruent is a condition in which emotions of target and context are the same. Incongruent is a condition in which emotions of target and context are not the same. Bold value indicates that the value is statistically significant.

## Discussion

Our study examined whether the congruency effect appeared differently depending on the type of emotion when individuals with BAP recognized facial expressions. This study had two major findings. First, the BAP group was not influenced by contextual information when they recognized the target emotion, whereas the control group was influenced by contextual information. Second, there was no significant difference in congruency effect depending on the type of emotion when the individuals with BAP recognized the emotion of the target.

The first major finding is that the individuals with BAP do not integrate contextual information with the target when they recognized facial expressions. The BAP group showed no difference in reaction time between the congruent and incongruent conditions, indicating that contextual information did not help them recognize the target emotion. Conversely, the control group performed faster in the congruent condition than in the incongruent condition, indicating that they could integrate the target with context and obtain help from the context. This finding supports the results of previous studies on WCCT. Evidence for WCCT in individuals with ASD has been found *via* neuroimaging studies as well as behavioral tasks (Happe, 1999; Ferstl and von Cramon, 2001; Mundy, 2003; Burnette et al., 2005). These results are also replicable in individuals with BAP, which means that individuals with BAP have a different cognitive pattern related to weak central coherence, resulting in a tendency to not holistically recognize emotional stimuli (Wright et al., 2008; Eto et al., 2014). These results are consistent with the expectations and results of this study. The BAP group was slower than the control group in emotion recognition, which could be related to their different cognitive patterns not being assisted by the context. However, the results should be interpreted carefully. A few studies reported that individuals with ASD, including BAP group, also have the global visual processing ability, and the ability just appears slower than typically developing individuals (Van der Hallen et al., 2015). This suggests that the BAP group, like the control group, can gaze at the context, and that they can also have a congruency effect. Indeed, when the analysis was conducted with emotions divided into negative and positive valences in our study (2 × 2 × 2 mixed-design ANOVA), there was no significant interaction effect on group × congruence, and there was a main effect of congruence and group. These results indicate that when the emotions were divided into valences, the congruency effect could appear in both the control group and the BAP group, and that the BAP group recognized the valence slowly compared to the control group. This result might have been obtained because it is easier to determine whether the target is positive or negative than to determine whether the target is fearful, sad, or happy, suggesting that the cognitive patterns of BAP could vary depending on task difficulty. Follow-up studies using eye trackers are needed to determine whether the congruency effect in the BAP group may appear differently when asked to classify the target as emotion and when asked to classify it as valence.

The second major finding is that the individuals with BAP would not integrate context information with the target regardless of the type of emotion, a finding inconsistent with the expectation of our study. This means that the type of emotion had no impact on the cognition pattern of BAP related to WCCT. This result can be explained using a model of face processing (Haxby et al., 2000). Based on the face-processing model, there are two stages of face processing: the core system and the extended system. The core system of face processing, the first stage, is related to visual analysis of faces. The extended system of face processing, the second stage, is related to processing information from a face, such as emotions or personal identity. Because the weak central coherence cognition style of individuals with BAP is related to the visual analysis method, local processing of the face in BAP might occur in the core system. On the other hand, emotional recognition from the face is undertaken in the extended system; therefore, emotion processing might be performed after the visual analysis related to weak central coherence. This may be the reason why the type of emotion does not affect the cognitive pattern of weak central coherence in the BAP group. That is, the cognitive pattern of WCCT had already appeared before the emotions of the target and context were recognized, so the BAP group processed only the emotion of the target. However, in the control group, they were able to gaze at both the target and the context in the first stage with a holistic cognitive pattern, and they processed both the emotion of the target and the context, resulting in a congruency effect.

Interestingly, both groups performed better when recognizing happy emotions than when recognizing fearful and sad emotions. Recent research may explain this result. Negative facial expressions can draw attentional resources more efficiently than happy facial expressions, allowing people to quickly detect potential threats (Vuilleumier and Huang, 2009). However, as hypervigilance toward negative faces makes it difficult to divert attention, motor responses are slower (Mirabella, 2018; Mancini et al., 2020, 2022; Mirabella et al., 2022). In our study, the facial stimuli to recognize were always task-relevant, and the BAP group also showed that happy emotions elicited faster responses than fear and sad emotions. This suggests that the BAP group used more attentional resources to assess negative expression because of their bias to process details, experienced hypervigilance from fearful and sad emotions, and resulted in the clicking of the button slowly.

In our study, differences in cognitive patterns between the BAP and control groups could be measured sensitively when the reaction time was used as a dependent variable. Accuracy was unable to sensitively differentiate the properties of the two groups; therefore, the two groups showed a similar level of accuracy and bias toward specific emotions for all the conditions. These results are similar to those of previous studies. People with autistic traits showed similar accuracy to the general population when they recognize basic emotions, although they showed slower reaction time than general population (Homer and Rutherford, 2008). However, some studies have revealed that individuals with autistic traits were less accurate when facial recognition tasks were more difficult (e.g., faces were presented with incongruent emotion label or shown for very brief durations) (Grossman et al., 2000; Clark et al., 2008). Based on the results of previous studies, the current study suggests that using complex emotions (e.g., guilt, shame, or envy) rather than basic emotions or proceeding under other complex conditions is more effective when further studies are conducted to confirm the decline in accuracy of individuals with BAP.

Importantly, three results were derived from the correlation analysis. First, the difference in reaction time between the BAP and control groups when recognizing facial emotions was related to the autistic traits assessed with the BAPQ and AQ. This result supports a previous research study showing that higher degree of autistic traits is related to impairments in recognizing facial emotions (Loth et al., 2018). Second, the relationship between behavioral parameters and autistic traits was more pronounced in the congruent condition than in the incongruent condition. This result is consistent with those mentioned above. Individuals with BAP could not be affected by context in congruent and incongruent conditions, so they showed no difference in reaction time between the two conditions. Contrary to the BAP group, the control group showed better performance in the congruent condition than in the incongruent condition because they could obtain compensation from the context representing the same emotion as the target. Since the difference in performance between the two groups was larger in the congruent condition, the correlation might also be stronger in the congruent condition. Third, although there was a positive correlation between reaction time and attention switching score, there was no correlation between reaction time and attention for detail score under the total, congruent, and incongruent conditions. This result suggests that the congruency effect does not appear in the BAP group not simply because they preferred detail but also because attention switching to other parts was not possible after gazing at the detail. Follow-up studies using an eye tracker are needed to clearly understand whether the cause of the congruency effect is attention switching or other factors.

The present study has several limitations. First, it confirmed that individuals with BAP tended not to integrate contextual information with the target because there was no significant difference between the congruent and incongruent conditions. The congruency effect could be investigated more explicitly if follow-up studies employ an eye tracker to verify the pattern of eye movements. It is necessary to accurately examine by eye-tracking research whether individuals with BAP react after staring at the target without looking at the context, whether they show slow recognition of emotions after looking at the context, or whether they slowly recognize emotions because of slow attention switching after looking at the context. Second, our study used fearful, sad, and happy emotions to identify the effect of context on the target along with the type of emotions in individuals with BAP. Although no significant interaction between types of emotions and the congruency effect was found in individuals with BAP, it remains necessary to conduct a study based on other basic or complex emotions. Third, this study excluded the combinations of fear and sadness from the incongruent condition. As we presented in the Supplementary material, the reaction time to recognize fearful target in happy or sad context exhibited a different pattern compared to the other conditions. Especially, the interquartile range and standard deviation of reaction time in this incongruent condition were much larger than those of the other conditions (Supplementary Figure 2 and Supplementary Table 4). These may be because the fearful face in sad contexts exhibited properties different from those of the fearful face in happy context. Therefore, we decided that considering the fearful or sad face presented in the sad or fearful context as an incongruent condition would not have a clear result and decided to exclude the combinations of fear and sadness from the analysis.

The present study provides evidence that individuals with BAP have cognitive patterns predicted by the WCCT, and that these patterns appear regardless of the type of emotion when recognizing facial emotion. These results have a research implication that the diminished performance in recognizing emotions observed in individuals with BAP can be attributed to their diminished ability to integrate the target stimuli with contextual information. These results suggest that the diminished ability of the BAP group to recognize emotions can be affected by their cognitive patterns related to weak central coherence. The current study also offers a clinical implication that some interventions are effective. For individuals with BAP, interventions involving eye feedback could modify their cognitive patterns, thereby permitting them to see the context surrounding the target and increasing their ability to recognize emotions. According to the behavioral plasticity theory, behavioral changes can cause changes in neuroanatomical properties (Mery and Burns, 2010). Therefore, repeated training of saccadic eye movement would improve eye movement performance and change the neurological properties of eye movement (Jamadar et al., 2015). If these cognitive patterns could be modified by training saccadic eye movements related to eye feedback, then the ability of individuals with BAP to recognize facial expressions would be improved.

## Data availability statement

The raw data supporting the conclusions of this article will be made available by the authors, without undue reservation.

## Ethics statement

The studies involving human participants were reviewed and approved by Institutional Review Board of Chung-Ang University. The patients/participants provided their written informed consent to participate in this study.

## Author contributions

W-JC: design the experimental task, participants’ data acquisition, and data analysis. Both authors: conception of the experiment data interpretation, drafting of the manuscript, revised the manuscript critically, and gave their final approval of the version to be published.
