# Supplementary material for: Diminished ability to integrate target stimuli with context during emotional recognition in individuals with broad autism phenotype
Source: Front Psychol. 2022 Oct 6;13:934385. doi: 10.3389/fpsyg.2022.934385 (PMC9583922; doi:10.3389/fpsyg.2022.934385)
Supplement: Supplementary file 1 [file Data_Sheet_1.docx]

Supplementary Material

# Supplementary Figures and Tables

## Supplementary Figures


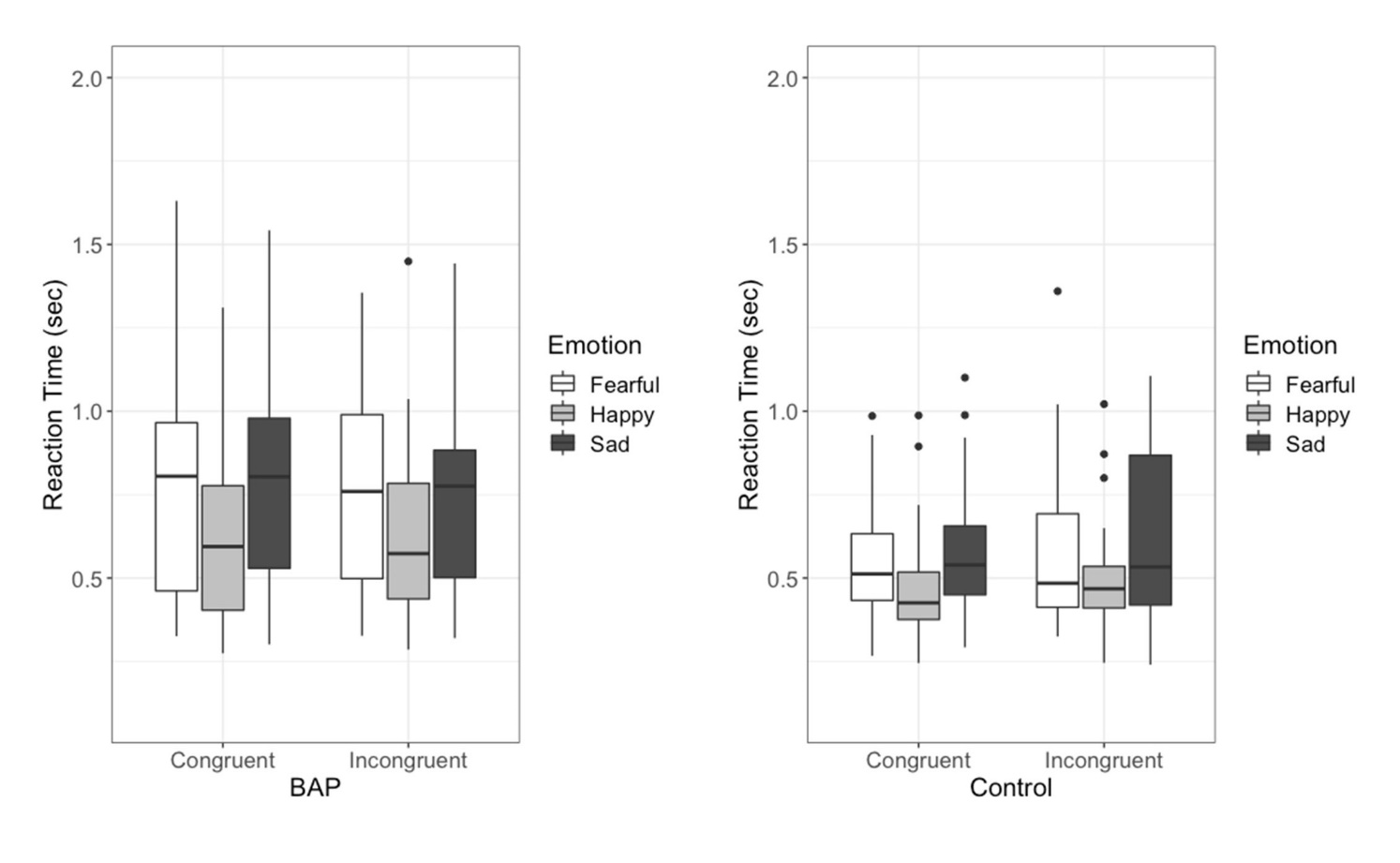


Supplementary Figure 1. Comparison of Reaction Time in BAP and Control Groups excluding Combinations of Fearful and Sad Emotions.

*Note*. This plot shows the results excluding the combinations of fearful and sad emotions from an incongruent condition; Congruent is a condition in which emotions of target and context are same; Incongruent is a condition in which emotions of target and context are not same; Box plots show median as the middle box line, first quartile (Q1) and third quartile (Q3) as box edges (denoting the interquartile range, IQR), whiskers as the minimum/maximum points and outliers based on thresholds < Q1 – 1.5(IQR) or > Q3 +1.5(IQR); BAP: Broad Autism Phenotype group, Control: Control group.


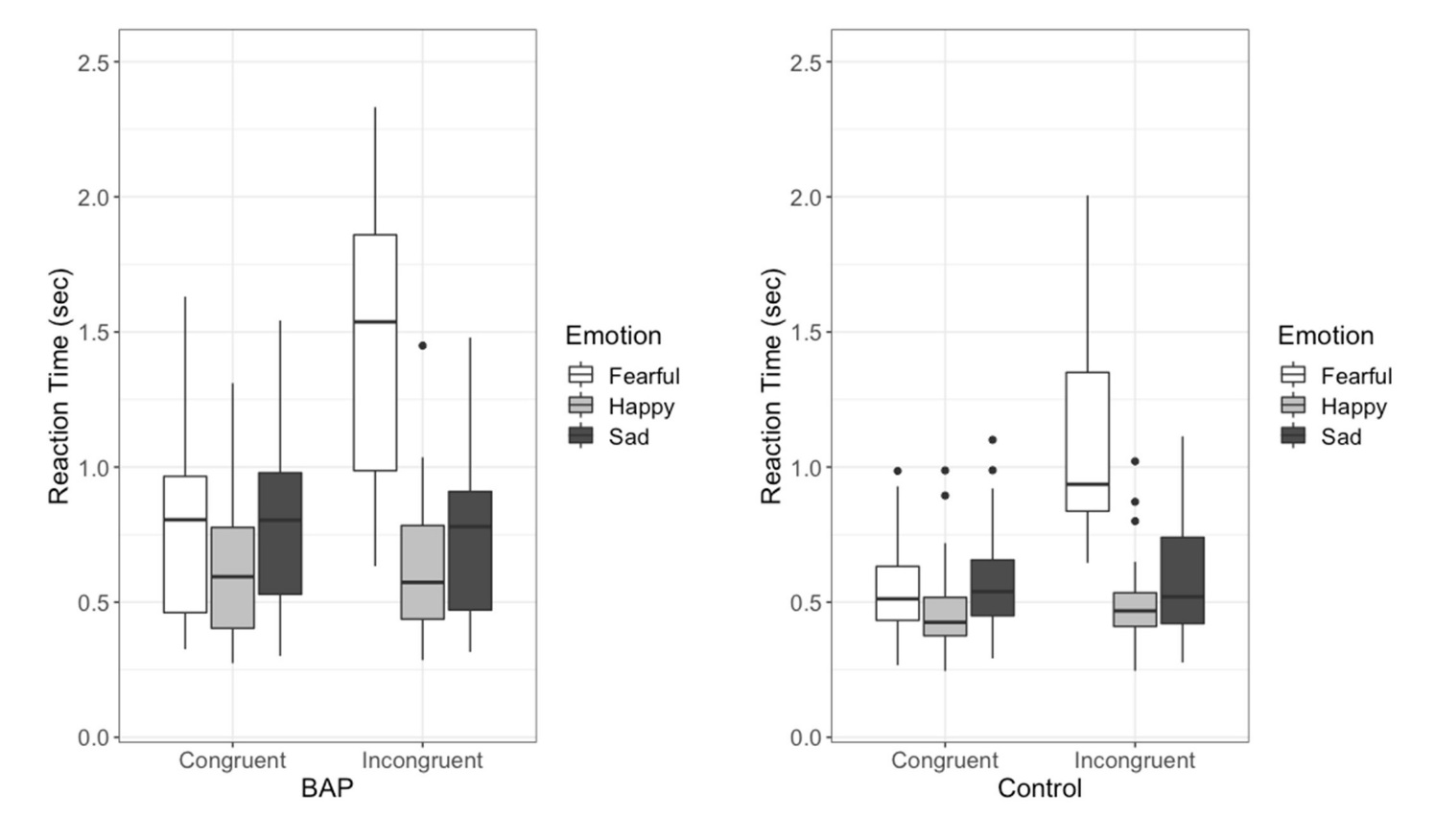


Supplementary Figure 2. Comparison of Reaction Time in BAP and Control Groups including Combinations of Fearful and Sad Emotions.

*Note*. This plot shows the results including the combinations of fearful and sad emotions as an incongruent condition; Congruent is a condition in which emotions of target and context are same; Incongruent is a condition in which emotions of target and context are not same; Box plots show median as the middle box line, first quartile (Q1) and third quartile (Q3) as box edges (denoting the interquartile range, IQR), whiskers as the minimum/maximum points and outliers based on thresholds < Q1 – 1.5(IQR) or > Q3 +1.5(IQR); BAP: Broad Autism Phenotype group, Control: Control group.

## Supplementary Tables

Supplementary Table 1. Images from IAPS, OASIS, and GAPED Used as Context

|  | IAPS | | | | OASIS | | | | GAPED | | | |
| --- | --- | --- | --- | --- | --- | --- | --- | --- | --- | --- | --- | --- |
| Fearful | 1114 | 1120 | 1300 | 1310 | - | | | | - | | | |
|  | 1321 | 1525 | 1726 | 1932 |  |  |  |  |  |  |  |  |
| Sad | - | | | | Dog 23 | Dog 24 | Dog 25 |  | A 007 | A 062 | A 072 | A 116 |
|  |  |  |  |  |  |  |  |  | A 127 |  |  |  |
| Happy | 1463 | 1710 |  |  | Dog 4 | Dog 6 | Dog 12 | Dog 13 | - | | | |
|  |  |  |  |  | Dog 19 | Dog 21 |  |  |  |  |  |  |

*Note. IAPS: International Affective Picture System; OASIS: Open Affective Standardized Image Set; GAPED: Geneva Affective Picture Database*

Supplementary Table 2. Arousal, Relevance, and Valence of Face and Context Stimuli

| Type | | Fearful (1) | Sad (2) | Happy (3) | Test Statistics  (*F*) | *p*-value | Post-hoc |
| --- | --- | --- | --- | --- | --- | --- | --- |
| Face | Arousal | 4.01 (0.934) | 3.88 (0.918) | 3.84 (1.43) | 0.092 | 0.912 | - |
|  | Relevance | 4.92 (0.654) | 5.18 (0.543) | 5.3 (0.621) | 1.496 | 0.236 | - |
|  | Positive Valence | 0.767 (0.477) | 0.578 (0.46) | 5.34 (0.672) | 367.4 | **<.001** | 3 > 1, 2 |
|  | Negative Valence | 4.48 (1.01) | 4.41 (1.25) | 0.308 (0.433) | 92.7 | **<.001** | 1, 2 > 3 |
| Context | Arousal | 4.46 (0.758) | 4.11 (0.837) | 4.22 (1.24) | 0.41 | 0.667 | - |
|  | Relevance | 4.88 (0.783) | 4.83 (0.858) | 4.95 (1.00) | 0.051 | 0.95 | - |
|  | Positive Valence | 0.771 (0.588) | 0.604 (0.442) | 4.86 (1.02) | 132 | **<.001** | 3 > 1, 2 |
|  | Negative Valence | 4.53 (0.769) | 4.74 (0.755) | 0.365 (0.504) | 154.8 | **<.001** | 1, 2 > 3 |

*Note. Mean (standard deviation). Using a 7-point Likert scale (0 = Not at all; 6 = Extremely)*

Supplementary Table 3. Summary of 2 (group) × 2 (congruence) × 3 (Emotion of target) Mixed-design ANOVA including Combinations of Fearful and Sad Emotions

| DV | IV | Test Statistics | | | |
| --- | --- | --- | --- | --- | --- |
|  |  | *df* | *F* | *p* | *BF_10_* |
| Reaction Time | Group × Congruence × Emotion | 2, 122 | 6.119 | **0.003** | 1.000 |
|  | Group × Congruence | 1, 61 | 1.601 | 0.211 | 0.522 |
|  | Group × Emotion | 2, 122 | 8.901 | **< .001** | 1.000 |
|  | Congruence × Emotion | 2, 122 | 278.005 | **< .001** | <.03 |
|  | Group | 1, 61 | 9.503 | **0.003** | 1.000 |
|  | Congruence | 1, 61 | 284.370 | **< .001** | 1.000 |
|  | Emotion | 2, 122 | 213.820 | **< .001** | 1.000 |
| Error Rate | Group × Congruence × Emotion | 2, 122 | 2.091 | 0.128 | <.03 |
|  | Group × Congruence | 1, 61 | 1.069 | 0.305 | <.03 |
|  | Group × Emotion | 2, 122 | 1.230 | 0.296 | 0.119 |
|  | Congruence × Emotion | 2, 122 | 0.182 | 0.834 | <.03 |
|  | Group | 1, 61 | 0.414 | 0.522 | <.03 |
|  | Congruence | 1, 61 | 3.498 | 0.066 | <.03 |
|  | Emotion | 2, 122 | 9.962 | **< .001** | 1.000 |
| Sensitivity | Group × Congruence × Emotion | 2, 122 | 0.371 | 0.691 | <.03 |
|  | Group × Congruence | 1, 61 | 0.078 | 0.781 | <.03 |
|  | Group × Emotion | 2, 122 | 1.015 | 0.365 | <.03 |
|  | Congruence × Emotion | 2, 122 | 0.013 | 0.987 | <.03 |
|  | Group | 1, 61 | 0.635 | 0.429 | 0.519 |
|  | Congruence | 1, 61 | 0.006 | 0.941 | 0.172 |
|  | Emotion | 2, 122 | 0.007 | 0.993 | 0.073 |
| Response Criteria | Group × Congruence × Emotion | 2, 122 | 0.706 | 0.496 | <.03 |
|  | Group × Congruence | 1, 61 | 0.237 | 0.628 | <.03 |
|  | Group × Emotion | 2, 122 | 0.999 | 0.371 | <.03 |
|  | Congruence × Emotion | 2, 122 | 0.016 | 0.984 | <.03 |
|  | Group | 1, 61 | 0.085 | 0.771 | 0.148 |
|  | Congruence | 1, 61 | 0.003 | 0.957 | 0.130 |
|  | Emotion | 2, 122 | 0.003 | 0.997 | 0.066 |

*Note.* *This table shows the results including the combinations of fearful and sad emotions as an incongruent condition; DV: Dependent variable; IV: Independent variable.*

Supplementary Table 4. Mean (SD) of Reaction Time (sec) under Each Emotion and Congruence Condition for Groups including Combinations of Fearful and Sad Emotions

| Facial Expression | Context | BAP (n = 32) | Control (n = 31) | Test Statistics | | | |
| --- | --- | --- | --- | --- | --- | --- | --- |
|  |  |  |  | *df* | *F* | *p* | *BF_10_* |
| Fearful | Con: Fearful | 0.780 (0.331) | 0.542 (0.165) | 1, 61 | 12.937 | **< .001** | 44.648 |
|  | Incon: Sad or Happy | 1.541 (0.584) | 1.141 (0.447) | 1, 61 | 9.289 | **0.003** | 11.153 |
| Sad | Con: Sad | 0.786 (0.289) | 0.571 (0.191) | 1, 61 | 10.965 | **0.002** | 21.234 |
|  | Incon: Fearful or Happy | 0.730 (0.268) | 0.592 (0.234) | 1, 61 | 4.782 | **0.033** | 1.869 |
| Happy | Con: Happy | 0.610 (0.246) | 0.471 (0.168) | 1, 61 | 6.784 | **0.012** | 4.174 |
|  | Incon: Fearful or Sad | 0.634 (0.263) | 0.489 (0.170) | 1, 61 | 6.703 | **0.012** | 4.041 |

*Note. This table shows the results including the combinations of fearful and sad emotions as an incongruent condition; Mean (standard deviation); BAP: Broad Autism Phenotype group, Control: Control group; Con: Congruent, Incon: Incongruent; Congruent is a condition in which emotions of target and context*

Supplementary Table 5. Summary of 2 (group: BAP, control) × 2 (congruence: congruent, incongruent) × 2 (valence of target: negative, positive) Mixed-design ANOVA

| DV | IV | Test Statistics | | | |
| --- | --- | --- | --- | --- | --- |
|  |  | *df* | *F* | *p* | *BF_10_* |
| Reaction Time | Group × Congruence × Valence | 1, 61 | 1.642 | 0.205 | > 30 |
|  | Group × Congruence | 1, 61 | 1.018 | 0.317 | 3.553 |
|  | Group × Valence | 1, 61 | 2.029 | 0.159 | > 30 |
|  | Congruence × Valence | 1, 61 | 0.000 | 0.984 | > 30 |
|  | Group | 1, 61 | 8.239 | **0.006** | 10.649 |
|  | Congruence | 1, 61 | 8.078 | **0.006** | 0.913 |
|  | Valence | 1, 61 | 70.534 | **< .001** | > 30 |
| Error Rate | Group × Congruence × Valence | 1, 61 | 1.084 | 0.302 | 0.191 |
|  | Group × Congruence | 1, 61 | 0.438 | 0.511 | 0.132 |
|  | Group × Valence | 1, 61 | 1.269 | 0.264 | 26.845 |
|  | Congruence × Valence | 1, 61 | 0.001 | 0.972 | > 30 |
|  | Group | 1, 61 | 0.138 | 0.712 | 0.263 |
|  | Congruence | 1, 61 | 2.808 | 0.099 | 1.302 |
|  | Valence | 1, 61 | 17.941 | **< .001** | > 30 |
| Sensitivity | Group × Congruence × Valence | 1, 61 | 0.011 | 0.916 | < 0.03 |
|  | Group × Congruence | 1, 61 | 0.522 | 0.473 | 0.030 |
|  | Group × Valence | 1, 61 | 0.008 | 0.931 | 0.020 |
|  | Congruence × Valence | 1, 61 | 0.000 | 0.998 | < 0.03 |
|  | Group | 1, 61 | 0.442 | 0.509 | 0.377 |
|  | Congruence | 1, 61 | 0.000 | 0.991 | 0.185 |
|  | Valence | 1, 61 | 0.000 | 0.999 | 0.327 |
| Response Criteria | Group × Congruence × Valence | 1, 61 | 0.010 | 0.921 | < 0.03 |
|  | Group × Congruence | 1, 61 | 0.003 | 0.955 | < 0.03 |
|  | Group × Valence | 1, 61 | 0.640 | 0.427 | < 0.03 |
|  | Congruence × Valence | 1, 61 | 0.000 | 0.999 | < 0.03 |
|  | Group | 1, 61 | 0.534 | 0.468 | 0.322 |
|  | Congruence | 1, 61 | 0.000 | 0.999 | 0.190 |
|  | Valence | 1, 61 | 0.000 | 0.990 | 0.187 |

*Note. DV: Dependent variable; IV: Independent variable.*

Supplementary Table 6. Mean (SD) and Main Effect of the Group, Congruence, and Valence Condition in Reaction Time and Error Rate

| DV | IV | Mean (SD) | | Test Statistics | | | |
| --- | --- | --- | --- | --- | --- | --- | --- |
|  |  |  |  | *df* | *F* | *p* | *BF_10_* |
| Reaction Time | Group | BAP | 0.691 (0.040) | 1, 61 | 8.239 | **0.006** | 10.649 |
|  |  | Control | 0.529 (0.040) |  |  |  |  |
|  | Congruence | Congruent | 0.599 (0.028) | 1, 61 | 8.078 | **0.006** | 0.913 |
|  |  | Incongruent | 0.620 (0.029) |  |  |  |  |
|  | Valence | Negative | 0.668 (0.031) | 1, 61 | 70.534 | **< .001** | > 30 |
|  |  | Positive | 0.551 (0.027) |  |  |  |  |
| Error Rate | Group | BAP | 5.261 (0.608) | 1, 61 | 0.138 | 0.712 | 0.263 |
|  |  | Control | 4.940 (0.618) |  |  |  |  |
|  | Congruence | Congruent | 4.811 (0.429) | 1, 61 | 2.808 | 0.099 | 1.302 |
|  |  | Incongruent | 5.389 (0.502) |  |  |  |  |
|  | Valence | Negative | 6.428 (0.635) | 1, 61 | 17.941 | **< .001** | > 30 |
|  |  | Positive | 3.773 (0.411) |  |  |  |  |

*Note. DV: Dependent variable; IV: Independent variable.*

Supplementary Table 7. Correlation Analysis between Error Rate and the Score of BAPQ and AQ

|  | | ER - Total | | | ER - Con | | | ER - Incon | | |
| --- | --- | --- | --- | --- | --- | --- | --- | --- | --- | --- |
|  |  | *r* | *p* | *BF_10_* | *r* | *p* | *BF_10_* | *r* | *p* | *BF_10_* |
| BAPQ | | -0.006 | 0.960 | 0.157 | 0.014 | 0.910 | 0.158 | -0.024 | 0.851 | 0.160 |
|  | Aloof | 0.046 | 0.720 | 0.167 | 0.072 | 0.573 | 0.167 | 0.018 | 0.888 | 0.159 |
|  | Pragmatic language | -0.001 | 0.991 | 0.157 | 0.018 | 0.891 | 0.157 | -0.018 | 0.888 | 0.157 |
|  | Rigid | -0.051 | 0.691 | 0.170 | -0.042 | 0.745 | 0.177 | -0.054 | 0.676 | 0.175 |
| AQ | | 0.023 | 0.856 | 0.160 | 0.023 | 0.858 | 0.158 | 0.021 | 0.869 | 0.158 |
|  | Social skill | 0.014 | 0.914 | 0.158 | 0.027 | 0.833 | 0.158 | 0.001 | 0.996 | 0.158 |
|  | Communication | 0.008 | 0.948 | 0.158 | -0.010 | 0.936 | 0.160 | 0.024 | 0.852 | 0.161 |
|  | Imagination | 0.087 | 0.496 | 0.197 | 0.125 | 0.328 | 0.21 | 0.045 | 0.728 | 0.164 |
|  | Attention for detail | 0.003 | 0.980 | 0.157 | 0.017 | 0.892 | 0.159 | -0.010 | 0.940 | 0.161 |
|  | Attention Switching | -0.014 | 0.992 | 0.158 | -0.056 | 0.662 | 0.179 | 0.024 | 0.852 | 0.165 |

*Note. ER: Error Rate; Con: Congruent, Incon: Incongruent; Congruent is a condition in which emotions of target and context are same. Incongruent is a condition in which emotions of target and context are not same; BAPQ: Broad Autism Phenotype Questionnaire; AQ: Autism Spectrum Quotient.*

Supplementary Table 8. Correlation Analysis between Sensitivity and the Score of BAPQ and AQ

|  | | Sensitivity - Total | | | Sensitivity - Con | | | Sensitivity - Incon | | |
| --- | --- | --- | --- | --- | --- | --- | --- | --- | --- | --- |
|  |  | *r* | *p* | *BF_10_* | *r* | *p* | *BF_10_* | *r* | *p* | *BF_10_* |
| BAPQ | | -0.093 | 0.474 | 0.195 | -0.063 | 0.625 | 0.173 | -0.110 | 0.395 | 0.214 |
|  | Aloof | -0.074 | 0.568 | 0.277 | -0.059 | 0.650 | 0.231 | -0.080 | 0.540 | 0.292 |
|  | Pragmatic language | -0.028 | 0.832 | 0.235 | -0.010 | 0.937 | 0.180 | -0.041 | 0.750 | 0.306 |
|  | Rigid | -0.140 | 0.277 | 0.157 | -0.094 | 0.467 | 0.158 | -0.166 | 0.198 | 0.158 |
| AQ | | -0.086 | 0.507 | 0.208 | -0.057 | 0.661 | 0.193 | -0.104 | 0.423 | 0.209 |
|  | Social skill | -0.132 | 0.308 | 0.221 | -0.094 | 0.467 | 0.184 | -0.152 | 0.237 | 0255 |
|  | Communication | -0.011 | 0.935 | 0.191 | -0.015 | 0.907 | 0.158 | -0.005 | 0.971 | 0.288 |
|  | Imagination | -0.212 | 0.098 | 0.361 | -0.171 | 0.185 | 0.483 | -0.227 | 0.077 | 0.238 |
|  | Attention for detail | 0.043 | 0.738 | 0.161 | 0.103 | 0.425 | 0.212 | -0.022 | 0.863 | 0.319 |
|  | Attention Switching | -0.023 | 0.856 | 0.166 | -0.042 | 0.747 | 0.162 | -0.002 | 0.987 | 0.229 |

*Note. Con: Congruent, Incon: Incongruent; Congruent is a condition in which emotions of target and context are same. Incongruent is a condition in which emotions of target and context are not same; BAPQ: Broad Autism Phenotype Questionnaire; AQ: Autism Spectrum Quotient.*

Supplementary Table 9. Correlation Analysis between Response Criteria and the Score of BAPQ and AQ

|  | | Response Criteria  - Total | | | Response Criteria  - Con | | | Response Criteria  - Incon | | |
| --- | --- | --- | --- | --- | --- | --- | --- | --- | --- | --- |
|  |  | *r* | *p* | *BF_10_* | *r* | *p* | *BF_10_* | *r* | *p* | *BF_10_* |
| BAPQ | | -0.046 | 0.723 | 0.674 | 0.008 | 0.953 | 0.505 | -0.074 | 0.569 | 0.364 |
|  | Aloof | -0.433 | 0.738 | 0.425 | 0.024 | 0.853 | 0.232 | -0.083 | 0.522 | 0.405 |
|  | Pragmatic language | -0.088 | 0.496 | 2.373 | -0.087 | 0.499 | 0.626 | -0.061 | 0.640 | 1.412 |
|  | Rigid | -0.005 | 0.972 | 0.284 | 0.065 | 0.615 | 0.692 | -0.058 | 0.653 | 0.162 |
| AQ | | -0.081 | 0.531 | 0.352 | 0.031 | 0.812 | 0.469 | -0.144 | 0.265 | 0.200 |
|  | Social skill | -0.068 | 0.601 | 1.074 | 0.031 | 0.810 | 1.551 | -0.124 | 0.336 | 0.318 |
|  | Communication | -0.230 | 0.073 | 0.362 | -0.165 | 0.200 | 0.227 | -0.208 | 0.105 | 0.331 |
|  | Imagination | -0.083 | 0.522 | 0.163 | -0.019 | 0.882 | 0.229 | -0.107 | 0.409 | 0.165 |
|  | Attention for detail | 0.107 | 0.408 | 0.159 | 0.190 | 0.139 | 0.208 | 0.008 | 0.954 | 0.221 |
|  | Attention Switching | 0.007 | 0.955 | 0.309 | 0.122 | 0.344 | 0.234 | -0.086 | 0.507 | 0.260 |

*Note. Con: Congruent, Incon: Incongruent; Congruent is a condition in which emotions of target and context are same. Incongruent is a condition in which emotions of target and context are not same; BAPQ: Broad Autism Phenotype Questionnaire; AQ: Autism Spectrum Quotient.*

Supplementary Table 10. Correlation Analysis between Congruency Scores and the Score of BAPQ and AQ

|  | | Congruency Score of RT | | | Congruency Score of Accuracy | | |
| --- | --- | --- | --- | --- | --- | --- | --- |
|  |  | *r* | *p* | *BF_10_* | *r* | *p* | *BF_10_* |
| BAPQ | | -0.287 | **0.022** | 2.012 | -0.058 | 0.649 | 0.174 |
|  | Aloof | -0.363 | **0.003** | 10.189 | -0.072 | 0.575 | 0.183 |
|  | Pragmatic language | -0.186 | 0.145 | 0.445 | -0.053 | 0.680 | 0.171 |
|  | Rigid | -0.217 | 0.088 | 0.654 | -0.027 | 0.836 | 0.161 |
| AQ | | -0.254 | **0.044** | 1.133 | 0.002 | 0.991 | 0.157 |
|  | Social skill | -0.127 | 0.322 | 0.254 | -0.037 | 0.775 | 0.164 |
|  | Communication | -0.181 | 0.156 | 0.420 | 0.052 | 0.684 | 0.171 |
|  | Imagination | -0.341 | **0.006** | 6.094 | -0.103 | 0.420 | 0.216 |
|  | Attention for detail | -0.131 | 0.305 | 0.263 | -0.040 | 0.757 | 0.165 |
|  | Attention Switching | -0.218 | 0.087 | 0.661 | 0.116 | 0.365 | 0.235 |

*Note. RT: Reaction Time; BAPQ: Broad Autism Phenotype Questionnaire; AQ: Autism Spectrum Quotient.*
